# Using the zebrafish model for Alzheimer’s disease research

**DOI:** 10.3389/fgene.2014.00189

**Published:** 2014-06-30

**Authors:** Morgan Newman, Esmaeil Ebrahimie, Michael Lardelli

**Affiliations:** School of Molecular and Biomedical Science, University of AdelaideSA, Australia

**Keywords:** zebrafish, Alzheimer disease, presenilins, gamma-secretase, amyloid beta-protein precursor, MAPT

## Abstract

Rodent models have been extensively used to investigate the cause and mechanisms behind Alzheimer’s disease. Despite many years of intensive research using these models we still lack a detailed understanding of the molecular events that lead to neurodegeneration. Although zebrafish lack the complexity of advanced cognitive behaviors evident in rodent models they have proven to be a very informative model for the study of human diseases. In this review we give an overview of how the zebrafish has been used to study Alzheimer’s disease. Zebrafish possess genes orthologous to those mutated in familial Alzheimer’s disease and research using zebrafish has revealed unique characteristics of these genes that have been difficult to observe in rodent models. The zebrafish is becoming an increasingly popular model for the investigation of Alzheimer’s disease and will complement studies using other models to help complete our understanding of this disease.

## ALZHEIMER’S DISEASE

Alzheimer’s disease (AD) is the most prevalent form of dementia. It was estimated in 2010 that 36 million people were living with AD and this figure is predicted to increase to 66 million by 2030 (World Alzheimer report’s, 2010). The main clinical feature of AD is progressive memory loss. However, AD can also be characterized by the impairment of speech and motor ability, depression, delusions, hallucinations, and aggressive behavior (Voisin and Vellas, 2009). Despite these recognizable behavioral differences it is actually difficult during the early stages of the disease to correctly diagnose someone as having AD (Blennow et al., 2006). There is significant neuronal loss in several brain regions in AD patients (Regeur et al., 1994; West et al., 1994). This is usually accompanied by extracellular aggregates of the amyloid-beta peptide and intracellular aggregates of the tau protein called neurofibrillary tangles (NFTs).

Alzheimer’s disease can be classed as familial (FAD, usually with onset <65 years) or sporadic (SAD, onset >65 years). We have acquired most of our knowledge on the pathogenesis of AD through studies of FAD mutations. FAD is attributed to mutations in three genes, *PRESENILIN 1* (*PSEN1*), *PRESENILIN 2* (*PSEN2*), and the *AMYLOID BETA A4 PRECURSOR PROTEIN* (*APP*). The PSEN proteins are essential for γ-secretase activity that cleaves transmembrane domain proteins within lipid bilayers (reviewed in McCarthy et al., 2009). The PSEN proteins and four other proteins comprise γ-secretase complexes. PSEN (1 or 2), nicastrin (NCT), anterior pharynx defective 1 (APH1a or APH1b), and presenilin enhancer 2 (PSENEN) form active γ-secretase complexes in cellular membranes. These complexes are responsible for the cleavage of a number of single-pass transmembrane proteins such as APP and NOTCH. APP is initially cleaved by α- or β-secretases to release APPα or APPβ fragments, respectively. The subsequent cleavage of APP by γ-secretase after β-secretase cleavage liberates amyloid-β (Aβ) peptides of various lengths. The longer Aβ-42 peptide is prone to aggregation and is suggested to form toxic oligomers and fibrils that eventually deposit as amyloid plaques in the brain. Deposits of plaques and NFTs are common occurrences in the brains of those with FAD.

The common, late onset form of AD (accounting for >90% of cases) occurs sporadically (Blennow et al., 2006). Sporadic AD (SAD) has a complex etiology and is associated with many risk factors including old-age and possession of the ε4 allele of the *APOLIPOPROTEIN E* (*APOE*) gene. Human genome wide association studies have revealed a number of possible loci associated with risk for SAD [reviewed by Rademakers and Rovelet-Lecrux, 2009). There are numerous other risk factors associated with an individual’s lifestyle that can also increase the likelihood of developing AD. These overlap considerably with the risk factors for cardiovascular disease (Kotze and van Rensburg, 2012) including hypertension, hypercholesterolemia and obesity (Martins et al., 2006). The molecular and cellular triggers for the onset of SAD are yet to be completely resolved, however, there have been many hypotheses suggested to explain how AD is initiated. Of the many hypotheses that have been postulated over the years, some that are gaining momentum attribute AD to vasculature dysfunction (Stone, 2008; Marchesi, 2011), oxidative stress (Nunomura et al., 2001), mitochondrial dysfunction (Selfridge et al., 2012) or hypoxia (Oresic et al., 2011).

The common observation of amyloid plaques in AD brains led to the formulation of the amyloid hypothesis in 1992 (Hardy and Higgins, 1992) that continues to dominate research thinking as a unifying hypothesis for FAD and SAD. The hypothesis suggests that toxic oligomerization of Aβ peptides is the initiating factor that triggers a cascade of subsequent cellular abnormalities such as inflammation and oxidative stress (Verdile et al., 2004). These secondary phenomena ultimately lead to neuronal dysfunction, degeneration and death. Although still widely accepted, doubts have arisen regarding its validity following several failed clinical trials of drugs intended to reduce Aβ levels (refer to http://www.alzforum.org regarding information on AD drugs in clinical trials).

Rodent models of AD have been exploited extensively and have given considerable insight into this disease. However, we still do not understand the events that trigger the neurodegeneration evident in AD. Research using the zebrafish model has revealed particular characteristics of the various genes implicated in AD that have been difficult to observe in other animal models (Liao et al., 2012; Newman et al., 2012). This review will focus on how the zebrafish has been used to study the genes and various cellular pathways implicated in FAD and SAD pathogenesis and will conclude by examining the limitations of the zebrafish and what the future may hold for use of this model in AD research.

## THE ZEBRAFISH MODEL

The zebrafish are a small, hardy freshwater fish native to India and often kept in home aquaria. They were originally used as a model organism for the study of vertebrate development. However, over the last decade the zebrafish model has become increasingly employed for investigating a wide variety of human diseases (Lieschke and Currie, 2007). The zebrafish has a number of characteristics that make it a versatile animal model. Although they lack the advanced cognitive behaviors evident in rodent models, their transparent embryos, rapid development *ex utero* and large reproductive capacity (100+ embryos per spawning) provide obvious advantages over mammalian models. Furthermore, multiple genes can be manipulated simply and effectively in the zebrafish at physiologically relevant levels (Newman et al., 2012), which cannot be currently achieved in rodent models. While, in general, rodents more closely model human physiology than fish, zebrafish nevertheless are vertebrates and so are more relevant to understanding human biology than invertebrate models as *Drosophila melanogaster* and *Caenorhabditis elegans*. Zebrafish embryos are particularly manipulable due to their large size, ready availability and the ability to exploit changes in their development for assay of particular gene activities (Nornes et al., 2009). Thus, zebrafish embryos can often present a felicitous vertebrate system in which to examine the molecular and cellular functions of genes implicated in AD.

The zebrafish genome is extensively annotated. The zebrafish (teleost-bony fish) evolutionary lineage separated from the human (tetrapod) lineage approximately 450 million years ago (Kumar and Hedges, 1998). Teleosts appear to have undergone an additional round of genome duplication since their separation from the tetrapod lineage followed by loss of many of the duplicated genes (Catchen et al., 2011). However, in most cases, zebrafish genes can be identified that are clear orthologs of human genes. Zebrafish possess genes orthologous to the human genes that are thought to play essential roles in AD. The *psen1* (Leimer et al., 1999) and *psen2* (Groth et al., 2002) genes are orthologs of human *PSEN1* and *PSEN2*, respectively, while the *appa* and *appb* genes are “co-orthologs” of human *APP* (Musa et al., 2001). Zebrafish hold orthologous genes for the components of the gamma-secretase complex, *PSENEN* (*psenen*; Francis et al., 2002; Campbell et al., 2006), *NCTN* (*ncstn*; Strausberg et al., 2002) and *APH1b* (*aph1b*; Francis et al., 2002). While orthologs of β-secretase (*BACE1* and *BACE2*) have also recently been identified in zebrafish: *bace1* (Moussavi Nik et al., 2012) and *bace2* (van Bebber et al., 2013). The *microtubule-associated protein tau* (*MAPT*) gene in humans encodes the tau protein and our laboratory identified co-orthologs of this gene in zebrafish, *mapta*, and *maptb* (Chen et al., 2009). Zebrafish also possess co-orthologs of *APOE*: *apoea* and *apoeb* (Babin et al., 1997; Woods et al., 2005). Genes arising by duplication can have overlapping functions. This can be disadvantageous when analyzing gene function as particular loss-of-function phenotypes may be obscured unless the function of both duplicate genes is blocked. Alternatively, duplicate genes can have partially non-overlapping functions and this can facilitate functional analysis as loss-of-function phenotypes may be restricted to particular cells or tissues.

Zebrafish are an advantageous model for genetic and molecular studies. Zebrafish embryos are genetically malleable by injection of morpholino antisense oligonucleotides, mRNA, transgenes and more recently by genome engineering systems (reviewed in Hwang et al., 2013; Schmid and Haass, 2013; Hisano et al., 2014). These technologies can make both subtle and drastic changes in gene expression with the effects observed in the developing transparent embryos. Morpholinos are designed to bind to particular sites in transcripts from a gene of interest. Binding of a morpholino can either block mRNA translation (knockdown) or interfere with correct splicing of exons (e.g., Draper et al., 2001; Nornes et al., 2008; Berger et al., 2011). Injection of sense mRNA can allow overexpression of a particular gene of interest. The effects of morpholino and mRNA injection generally only persist during embryogenesis (2–3 days post-fertilization). Transgenic zebrafish can be generated using efficient vectors such as the Tol2 transposase system (Kawakami et al., 2000) to insert genes under the control of tissue specific promoters. Conditionally expressed transgenics can also be generated using the Cre/loxP (Hans et al., 2009) and GAL4-UAS (Halpern et al., 2008) systems for gene function analysis at particular time points. The absence of technology available to generate targeted mutations in the zebrafish genome was previously a disadvantage of the zebrafish model relative to rodents. In recent times however, zinc finger nucleases (ZFNs), transcription activator-like effector nucleases (TALENS) and the type II prokaryotic CRISPR (clustered regularly interspaced short palindromic repeats)/Cas systems have been developed for targeted modification of gene sequences in the zebrafish genome (Hwang et al., 2013; Schmid and Haass, 2013).

**FIGURE 1:**
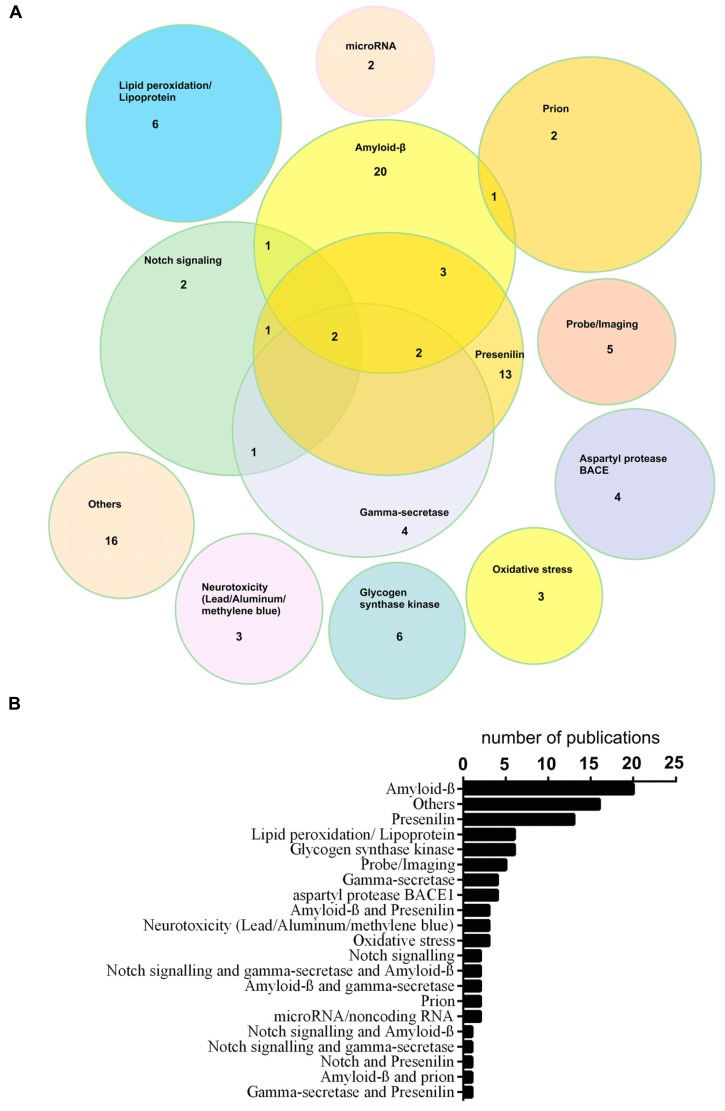
**Text mining analysis of the scientific literature to detect papers on the application of Zebrafish in Alzheimer’s disease research.** The PubMed database was mined for zebrafish and Alzheimer’s disease using the search term, “zebrafish+alzheimer’s disease,” by the MedScan Reader 5 tool implemented in the Pathway Studio 9 Package (Nikitin et al., 2003b; Novichkova et al., 2003a). MedScan Reader 5 collects information and processes undertaken data using natural language processing (NLP). The language interprets these to logical concepts and extracts functional relationships between proteins, small molecules, and cellular processes (Nikitin et al., 2003b; Novichkova et al., 2003a). 119 publications were found. Abstracts of these publications were studied and review papers were removed such that 98 papers remained. **(A)** Venn diagram and **(B)** bar graph showing numbers of research papers in different areas. A concentration of research on Amyloidβ and Presenilin function is apparent.

A search of the available scientific literature in the PubMed database using the term “zebrafish + alzheimer’s disease” revealed 119 publications with 98 being original research papers (23rd May, 2014). **Figure 1** displays the research areas in which these papers have been published. There is an obvious concentration of research on Aβ and Presenilin function in zebrafish.

## USING ZEBRAFISH TO INVESTIGATE GENES MUTATED IN FAD

### THE PRESENILINS

The genes implicated in FAD have been extensively studied over the last 25 years, although our understanding of their normal functions is still far from complete. The *PSEN* genes play important roles in development. The zebrafish (as well-characterized model for studies of developmental biology) has been employed to investigate the normal functions of these genes. The *psen1* and *psen2* genes are ubiquitously expressed in zebrafish embryos (Leimer et al., 1999; Groth et al., 2002), implying a functional importance of these genes during development. Injection of morpholinos to knockdown *presenilin* gene expression revealed similar and dissimilar phenotypes compared to Presenilin knockout mice (*Psen1*^-^^/^^-^). *Psen1*^-^^/^^-^ mice die during development (Shen et al., 1997). To overcome this, conditional *Psen1* knockout mouse models have been developed (reviewed in van Tijn et al., 2011). Zebrafish embryos injected with a *psen1* translation-blocking morpholino are viable and have similar phenotypes (Nornes et al., 2003; Campbell et al., 2006; Nornes et al., 2008) to *Psen1*^-^^/^^-^ mice such as aberrant somite formation and Notch signaling defects (Shen et al., 1997; Wong et al., 1997) Interestingly, a zebrafish mutant (discovered in a TILLING screen) lacking Psen1 activity is viable (Sundvik et al., 2013). These mutant fish have been used to study the brains histaminergic system (Sundvik et al., 2013). This system is essential in mediating cognitive functions affected in AD. Analysis of histamine neuron numbers in the *psen1*^-^^/^^-^ zebrafish brains revealed that *psen1* in zebrafish is a regulator of histaminergic neuronal development (Sundvik et al., 2013). These mutant fish will be a valuable tool for further analysis of *psen1* function in zebrafish.

Blockage of Psen2 protein translation in zebrafish embryos (Nornes et al., 2008, 2009) appears to have major effects on Notch signaling in comparison to *Psen2*^-^^/^^-^ mice which are viable and show only a minor phenotype (Herreman et al., 1999). Furthermore, loss of Psen2 expression affects the production of dorsal longitudinal ascending (DoLA) interneurons in the developing spinal cord of zebrafish larvae (Nornes et al., 2009). As loss of Psen1 does not affect DoLAs this observation provides an *in vivo* assay for Psen2 function. This assay was used to demonstrate a functional interaction between Psen1 and Psen2 in zebrafish (Nornes et al., 2009).

In the absence of technology available to analyze *PSEN* FAD missense mutations in zebrafish, our laboratory previously attempted to model *PSEN1* splicing mutations by injecting morpholinos that target splice acceptor sites of the orthologous *psen1* gene. We attempted to cause exon skipping to exclude exon 8 or 9 from zebrafish *psen1* transcripts to mimic the effects of the human *PSEN1 L271V* and Δ9 mutations, respectively,. However, instead of the (at that time) expected exon exclusion, injection of morpholinos blocking splice acceptor sites lead to the failure of introns 7 and 8 to be correctly spliced out of transcripts (Nornes et al., 2008). The inclusion of intron sequence lead to premature truncation of the open reading frame (ORF) after exons 6 and 7, respectively. It appeared that the aberrant transcripts induced by morpholino injection were able to evade nonsense-mediated decay and be translated into truncated protein molecules. Assays developed in our laboratory (discussed in the next section) demonstrated that truncation of the zebrafish *psen1* transcript ORF after exons 6 and 7 had potent dominant negative effects on Psen1 activity and could also suppress Psen2 activity (Nornes et al., 2008). These putative truncated proteins were subsequently expressed in embryos by injection of synthetic mRNAs. Injection of mRNAs coding for proteins truncated after exon 6- or 7-derived sequence had the same dominant effect on Psen1 activity as the morpholino-induced aberrant *psen1* transcripts. Recently we published results where we expanded the original study to analyze a series of *psen1* ORF truncations (i.e., after exon 4, 5, 6, 7, 8; Newman et al., 2014). We found differential, dominant activation of Notch signaling and APP cleavage amongst the different truncations of Psen1. Interestingly, similar effects were also observed after injection of synthetic mRNAs encoding equivalent truncations of human PSEN1. This supports the evolutionary conservation of function of these truncated proteins. A particularly important discovery from this study was that the activity of a form of Psen1 truncated after exon 4 sequence of the ORF) cannot be observed in the absence of normal endogenous Psen1. This implies that mutant forms of PSEN1 require endogenous normal PSEN1 to exert their effects. A similar observation was made recently by Heilig et al. for various FAD mutant forms of the PSEN1 protein in mouse embryo fibroblasts (Heilig et al., 2013).

### OTHER COMPONENTS OF THE γ-SECRETASE COMPLEX

There has been limited functional analysis of the zebrafish genes ortholgous to the non-PRESENILIN components of the γ-secretase complex. Blockage of translation of Psenen or Aph1b proteins in zebrafish causes phenotypes including defective somitogenesis and reduced neuron formation as expected for loss of Notch signaling (that requires γ-secretase activity; Campbell et al., 2006). Furthermore, blockage of Psenen translation leads to destabilization of Psen1 protein and causes a much greater induction of apoptosis in developing embryos than blocking translation of Psen1, Psen2, or Aph1b (Campbell et al., 2006). Simultaneous inhibition of p53 translation was able to block the induction of apoptosis (Campbell et al., 2006).

### THE AMYLOID-BETA PRECURSOR PROTEIN

The zebrafish *APP* “co-orthologs,” *appa* and *appb* have widespread and overlapping expression from mid-gastrulation in the developing embryo (Musa et al., 2001). At 24hpf both genes are expressed in the developing forebrain and other tissues with only *appb* expressed in the spinal cord (Musa et al., 2001). In a zebrafish study by Lee and Cole a section of *appb* regulatory sequence was fused to GFP (Lee and Cole, 2007). Dissimilar to other expression studies, they observed expression of *appb* in the developing vasculature. More recently, Liao et al. (2012) isolated transposon gene trap integrations that contained RFP in the *appa* gene and in the closely related a*myloid beta precursor-like protein 2 gene (aplp2)*. The gene traps caused fusions to RFP of the extracellular domains of both of the encoded proteins. The fusion proteins of these genes were accumulated in the vasculature. However, they could not detect the transcripts of these genes in the endothelial cells of the vasculature. Instead, transcripts were detected in neuronal cells. This suggests that these proteins are synthesized in neuronal cells and then accumulate in the vasculature.

Translation blocking morpholinos have also been employed to investigate the function of the Appa and Appb proteins (Joshi et al., 2009). Inhibition of Appa had little effect on the developing embryo while Appb translation inhibition resulted in defective convergent extension cellular movements and a reduced body length. These defects could be rescued by injecting Appb deficient embryos with mRNA coding for human APP. The rescue by human APP was more effective than injection of an mRNA encoding a human *APP* FAD mutant (the APP Swedish double mutation *K595N* and *M596L*). Loss of Appb activity has also been shown to cause defective neural development (Song and Pimplikar, 2012) including defective axonal out-growth patterning and synapse formation (Abramsson et al., 2013). In the study by Song and Pimplikar, only full-length human APP but not truncated forms could rescue the neuronal defects, revealing that both intracellular and extracellular domains of human APP are required for normal function (Song and Pimplikar, 2012). These studies demonstrate that zebrafish embryos can be exploited for the analysis of different mutant forms of human *APP*.

### AMYLOID-BETA FUNCTION AND TOXICITY STUDIES IN ZEBRAFISH

Although Aβ was discovered over 25 years ago (Goate and Hardy, 2012) and many studies have examined its claimed toxicity, the non-pathological functions of the Aβ peptide are still poorly understood. A study by Cameron et al. (2012) demonstrated that high levels of Aβ can increase cerebrovascular branching in the developing zebrafish hindbrain. They then completed a follow up study to determine whether lowering Aβ levels would have the opposite effect (Luna et al., 2013). From 2 days post fertilization (dpf) APP-deficient (by morpholino injection) and Aβ deficient (endogenous Aβ production blocked using a β-secretase inhibitor) larvae presented with cerebrovascular defects. Interestingly, these defects could be rescued by treating the embryos with the human Aβ-42 peptide but not the shorter cleavage product of APP named p3 (that arises from cleavage of APP by α-secretase and γ-secretase). These results suggest that Aβ may play a role in maintaining normal cerebrovascular function. This interesting finding using the zebrafish model provided further evidence that caution should be taken when treating AD with drugs designed to inhibit Aβ production such as BSIs and γ-secretase inhibitors (GSIs).

As mentioned above the amyloid hypothesis of AD proposes that aggregations of Aβ peptides are toxic. Therefore, there has been a considerable effort put towards developing and discovering Aβ-lowering compounds to counter the toxicity. Inhibition of β-secretase, also known as beta-site APP cleaving enzyme (BACE1), is of prime interest for the development of amyloid-lowering drugs. BACE1 has become an attractive target for drug development as, in comparison to the lethality observed in *Psen1* null mice, *Bace1* knock-out mice are viable (Roberds et al., 2001; Luo et al., 2003) and have subtle phenotypes (Harrison et al., 2003; Willem et al., 2006). Furthermore no phenotypes have been reported for knockout of the *Bace1* homolog, *Bace2* and deletion of *Bace2* in mice did alter Aβ generation (Dominguez et al., 2005). Recently, *bace1* and *bace2* loss-of-function mutations have been introduced into the zebrafish genome by genome editing using ZFNs (van Bebber et al., 2013). *bace1* mutants had an increased number of mechanosensory neuromasts and, similar to observations in the mouse, *Bace1* knockout caused a decrease in myelination. *bace2* mutants had a distinct melanocyte migration phenotype that was not observed in the *bace1* mutants. There was no effect on myelination or neuromasts in the *bace2* mutants. The phenotypes observed in the single mutants were not enhanced further in the Bace1/2 double knockout. Together the data from this study suggests that, in zebrafish, Bace1 and Bace2, have distinct non-redundant physiological functions. These specific phenotypes observed in zebrafish *bace1* and* bace2* mutants have provided important information on BACE1 and BACE2 function and support that zebrafish can be a useful *in vivo* system for determining, e.g., whether BACE1 inhibitors also inhibit BACE2 function and vice versa. *In vivo* evaluation of BACE inhibitors is integral for establishing these drugs as a therapeutic option for AD.

The zebrafish is an excellent system for the screening of chemical libraries. Embryos and larvae placed into microtiter plates can be treated with various chemicals in their aqueous support medium. This strategy can be employed to reveal therapeutic compounds for various disease states modeled in the fish. The only published study that has generated a transgenic Aβ toxicity model in zebrafish involved fusing the human Aβ-42 sequence to the promoter of the *mitfa* (*nacre*) gene (Newman et al., 2010). This would drive expression of human Aβ-42 specifically in the melanin-containing pigment cells (melanocytes) of the zebrafish in the hope that it would produce an easily identifiable disrupted pigmentation pattern phenotype without being lethal to the zebrafish larvae. A disrupted pattern did become evident in the adult fish. However, unfortunately it only became apparent after 16 months which is too late for drug screening and was too late for breeding the old, infertile fish. Aβ toxicity can also be analyzed in zebrafish simply by exposing embryos to amyloid-beta in their supportive aqueous environment. Treatment of embryos with 2.5 μM Aβ-40 caused defective development including that of the vasculature and also accelerated cell senescence (Donnini et al., 2010). This system could be expanded to test Aβ-42 (more aggregative form) and also to find compounds that ameliorate these observed defects.

γ-secretase inhibitors (GSIs) have been investigated as a therapeutic option to inhibit the production of Aβ peptides. However, the use of GSIs comes with the potential side affects of also affecting Notch signaling. Testing the toxicity of GSIs on zebrafish has revealed important information on how Notch related pathways are adversely affected by GSIs (Yang et al., 2010).

## ASSAYS FOR AD-RELEVANT CELLULAR PATHWAYS AND PROCESSES IN ZEBRAFISH

### γ-SECRETASE ACTIVITY ASSAYS

There are a number of cellular pathways and processes that are aberrant in AD. The zebrafish are a useful system for investigating molecular events such as γ-secreatse activity and autophagy that have been implicated in AD pathogenesis.

There are over 70 proteins that are known to be substrates of γ-secreatse activity (Lleo and Saura, 2011). In zebrafish, γ-secretase activity was initially analyzed by observing changes in the expression of genes known to be downstream targets of Notch signaling such as *hairy-related 6* (Bernardos et al., 2005; Campbell et al., 2006; Arslanova et al., 2010) and *neurogenin* (Campbell et al., 2006). However, gene expression can be under different transcriptional control in different regions of the embryo (Campbell et al., 2006; Arslanova et al., 2010). Therefore, using whole embryos in quantitative PCR analysis is not informative to assess changes in gene expression in response to various factors. To overcome this, changes in gene expression in a particular region of the embryos have been assessed by whole-mount *in situ* transcript hybridization (Campbell et al., 2006; Nornes et al., 2008; Arslanova et al., 2010). However, this is not normally used as a quantitative technique since many variables can influence the strength of the staining signal such as fixation conditions and incubation times. Consequently, use of this technique to assay relative differences in Notch signals under various treatments requires stringent controls.

Recently, the first assay to assess directly γ-secretase cleavage activity was developed by Wilson and Lardelli (2013). α- and β-secretase cleavage of Appa provide substrates for subsequent γ-secretase cleavage. Unfortunately these cleaved forms of Appa cannot be detected in zebrafish embryos prior to 48 hpf which currently makes monitoring endogenous Appa cleavage in manipulated zebrafish embryos difficult. Therefore, a fragment of Appa equivalent to the membrane-embedded remnant of APP following β-secretase cleavage was fused to GFP and expressed transiently in zebrafish embryos by the use of Tol2 vector transgenesis system. This construct is co-expressed with a set ratio of free GFP (for signal normalization). Western immunoblotting is then used to assess the ratio of the Appa:GFP substrate to free GFP (the γ-secretase cleavage product itself is too unstable to be observed). Once an Appa:GFP/ free GFP ratio is determined for a protein sample from a pool of manipulated embryos (e.g., drug treatment, morpholino or mRNA injection) it can then be compared to control embryos to determine how that particular manipulation is affecting γ-secretase cleavage activity.

### ASSAYING PROTEIN DEGRADATION PATHWAYS IN ZEBRAFISH

Excess or aberrant cellular proteins can be degraded by the ubiquitin-proteasome system (UPS). Since protein aggregation is implicated in many neurodegenerative diseases (Ross and Poirier, 2004) it is unsurprising that problems with UPS function have been implicated in neurodegenerative disease such as AD (West et al., 1994; Nikitin et al., 2003a; Novichkova et al., 2003b) and Parkinson’s disease (Olanow and McNaught, 2006). Zebrafish have been used to investigate UPS function in Parkinson’s disease (Regeur et al., 1994); however, no analyses in zebrafish have yet examined the UPS with respect to AD.

Autophagy is an important mechanism required for the degradation of dysfunctional and unwanted cellular components (including incorrectly folded and aggregated proteins) through the actions of lysosomes. Indeed, autophagy has been identified as a pathway for the degradation of accumulated Aβ peptides (Nilsson et al., 2013). Recently, the Presenilin proteins were suggested to have a major role in autophagy with FAD mutations in human PSEN1 inhibiting this function (Lee et al., 2010). These authors presented evidence showing that PSEN1 acts as a chaperone in the ER for a transmembrane protein required for acidification of the lysosomes, the v-ATPase V0a1 subunit [Lee et al., 2010; however, other reports have disputed this finding (Coen et al., 2012; Zhang et al., 2012)]. They also demonstrated that this function of PSEN1 is dependent on the full-length PSEN1 holoprotein rather than the endoproteolysed form that is active in the γ-secretase complex. Furthermore, a γ-secretase inhibitor and loss of another γ-secretase complex component (NCT) had no effect on autophagy (Lee et al., 2010) suggesting that this function of PSEN1 is independent from its function in γ-secretase complexes.

Autophagy can be analyzed in zebrafish by observing induction of the LC3-II protein by western immunoblotting using a human antibody against LC3 that cross-reacts with zebrafish Lc3 (He et al., 2009; He and Klionsky, 2010). Transgenic zebrafish have also been developed that express GFP fused to Lc3 (He et al., 2009). As Presenilin protein expression can easily be manipulated in the zebrafish these autophagy assay are a useful tool for further investigation of the involvement of the Presenilins in autophagy.

## USING THE ZEBRAFISH TO INVESTIGATE OTHER ASPECTS OF ALZHEIMER’S DISEASE ETIOLOGY

### ANALYSIS OF HYPOXIA IN THE ZEBRAFISH

There is accumulating evidence suggesting that hypoxia is an important initiating factor in the pathogenesis of AD. Under hypoxic conditions the electron transport chain in the mitochondria increases free radical production that leads to increased oxidative stress (Bell et al., 2007). Biomarkers of hypoxia can differentiate between people with mild cognitive impairment that progress to AD and those who do not (Oresic et al., 2011). The risk factors for cardiovascular disease and AD are similar (Kotze and van Rensburg, 2012) and it is anticipated that vasculature problems would affect oxygenation of the brain. Interestingly, Aβ levels in serum have been shown to be elevated after cardiac arrest (Zetterberg et al., 2011).

Zebrafish are an advantageous system for analysis of the effects of hypoxia on various biological functions. Zebrafish embryos and adults can be exposed to real hypoxia by depleting their water environment of oxygen or to chemical mimicry of hypoxia through, e.g., sodium azide treatment (Moussavi Nik et al., 2011). Similarly to what is observed in humans (Lukiw et al., 2001; Pluta, 2005; De Gasperi et al., 2010; Zhang and Le, 2010), hypoxia upregulates *psen1*, *psen2*, *appa*, *appb*, and *bace1* in zebrafish adult brain and larvae (Moussavi Nik et al., 2012). This suggests that Aβ is produced as a protective response to hypoxia in both human and zebrafish cells – a response conserved over 450 Mya of evolutionary time. Note, however, that while all the enzymes required to cleave Aβ from Appa and/or Appb are present in zebrafish, the existence of Aβ itself has not yet been directly demonstrated (e.g., through immunoblotting or mass spectrometry). The study by Moussavi Nik et al. (2012) also demonstrated that, unlike in mammals, F2-isoprostanes are not a good marker of oxidative stress in zebrafish and that the upregulation of *catalase* gene expression can be a better alternative marker for demonstration of oxidative stress in zebrafish (Jin et al., 2011; Tseng et al., 2013).

### APOE

The APOE ε4 allele has been identified has the main genetic risk factor for SAD. APOE is important for clearance of amyloid-beta from the brain (Huang and Mucke, 2012), while the AD risk-associated ε4 allele has been shown to impair the clearance of Aβ (Deane et al., 2008) and, more recently, to affect the integrity of the blood–brain barrier (Bell et al., 2012). There has been little research investigating APOE function in zebrafish. Expression studies of *apoea* (Raymond et al., 2006) and *apoeb* (Pujic et al., 2006) revealed expression in the developing retina and yolk syncytial layer. Furthermore, *apoeb* has also been observed in microglial cells (Veth et al., 2011), developing fins and epidermis (Monnot et al., 1999; Tingaud-Sequeira et al., 2006), regenerating fin tissue (Monnot et al., 1999), macrophages (Lien et al., 2006), liver, intestine, and ovary (Levi et al., 2012).

## MAPT

The MAPT is the main component of the NFTs found in AD brains. Various dysfunctions of the tau protein are found in other neurodegenerative disorders such as frontotemporal dementia (FTD), corticobasal degeneration and progressive supranuclear palsy (Pittman et al., 2006). Diseases with tau-like pathology are collectively termed “tauopathies.” A review by Bai and Burton (2011) discussed how the zebrafish has been used to investigate these diseases. A number of MAPT protein isoforms exist as a result of alternative splicing of *MAPT* transcripts. These isoforms can be classified into two groups, 3R or 4R, depending on the number of tubulin-binding motifs. It appears that an overall one-to-one ratio of 3R to 4R transcripts is required for normal functioning of the MAPT protein in the brain (Goedert and Spillantini, 2006). In most tauopathies this ratio is found to be changed (Goedert and Spillantini, 2006) and altered splicing of *MAPT* is also suggested to occur in AD brains (Conrad et al., 2007).

Transgenic zebrafish expressing human *MAPT* were generated and investigated prior to identification of the zebrafish ortholog(s) of *MAPT*. In these studies human *MAPT* was specifically expressed in zebrafish CNS neurons (Bai et al., 2007; Paquet et al., 2009). Bai et al. used the promoter of the *enolase 2* gene to drive the expression of MAPT 4R in zebrafish neurons at approximately eightfold higher levels than what is observed in human brain. This resulted in accumulations of tau protein (resembling NFTs) in the zebrafish brain. In the study by Paquet et al., the *HuC* promoter was employed to drive expression of a Gal4:VP16 fusion protein in neurons. This protein was then bound to UAS sites in a bidirectional promoter transcribing the DsRed fluorescent marker protein gene and a mutant form of human MAPT associated with FTD, *TAU-P301L*. The transgenic zebrafish larvae showed biochemical changes consistent with those observed in human tauopathies. However, it should be noted that no comparisons of phenotype were made between the non-mutant and mutant forms of human *MAPT* in the zebrafish. Furthermore, the expression levels of the transgenes relative to the endogenous zebrafish *mapt* genes were not assessed in these studies. Despite these limitations these transgenic zebrafish models provide a useful system to investigate whether chemical inhibitors can modulate the observed tauopathy-associated changes.

The zebrafish “co-orthologs” of the human *MAPT* gene, *mapta* and *maptb* have similar but not completely overlapping patterns of expression in developing embryos (Chen et al., 2009). They are both predominantly expressed in the developing CNS while only *maptb* has strong expression in the trigeminal ganglion and dorsal sensory neurons of the spinal cord. *Mapta* is spliced into 4R–6R isoforms while *maptb* is spliced mainly into 3R isoforms. This expression is in contrast to mouse *Mapt* which is mainly expressed as 3R in the brain (and hence may not be a good model of MAPT function for human pathologies). Manipulation of the expression of the zebrafish *mapt* isoforms may therefore be advantageous for understanding the function of 3R and 4R MAPT and the role(s) the 3R:4R ratio plays in pathogenesis.

## THE FUTURE FOR MODELING ALZHEIMER’S DISEASE IN ZEBRAFISH

The zebrafish is rapidly emerging as an attractive model for AD research. They are an ideal model for drug testing prior to clinical testing in rodents. However, there are still aspects of this model that require better understanding. For the zebrafish system to be used to model aspects of AD pathobiology we need to better understand zebrafish brain structure and function and also gain a deeper understanding of adult zebrafish brain physiology. Work so far has revealed that the zebrafish brain does have a reasonable level of conservation of basic structure when compared to mammals as well as similar neuroanatomical and neurochemical pathways to those that play roles in human disease (reviewed in Santana et al., 2012). We have revealed various aspects of presenilin gene biology using the zebrafish that would otherwise be difficult to observe/analyze in other models. However, to analyze effectively future transgenic and mutant zebrafish models of AD we need to strengthen our understanding of the functions in zebrafish of some of the orthologs of the key genes implicated in human AD pathogenesis such as MAPT and APOE.

Whether the zebrafish can be employed to model a late-onset disease like AD is debatable since zebrafish have a profound capacity for regeneration and this must impact on the development of neurodegenerative phenotypes. Neurogenesis in the adult zebrafish brain is much more abundant than is observed in mammals (Kizil et al., 2012) consequently making analysis of neuronal loss difficult. Despite these limitations the recent availability and feasibility of using genome editing technologies presents an exciting opportunity to develop zebrafish genetic models of neurodegenerative diseases such as AD. ZFNs, TALENs and CRISP/Rs have been validated for use in the zebrafish [reviewed by Hwang et al., 2013; Schmid and Haass, 2013) and it is inevitable that FAD mutations will be introduced into zebrafish FAD gene orthologs.

Animal models are a useful tool in investigating the causes and pathologies of human diseases. Obviously such models can never reflect the complete pathology that is observed in human cases. The complexity of the human brain makes AD a particularly difficult disease to model in animals. However, by using a number of different models including the zebrafish, we can exploit the unique characteristics of each to unravel the molecular basis of this disease.

## Conflict of Interest Statement

The authors declare that the research was conducted in the absence of any commercial or financial relationships that could be construed as a potential conflict of interest.
